# Recent Advances in the Application of the Antimicrobial Peptide Nisin in the Inactivation of Spore-Forming Bacteria in Foods

**DOI:** 10.3390/molecules26185552

**Published:** 2021-09-13

**Authors:** Christian Anumudu, Abarasi Hart, Taghi Miri, Helen Onyeaka

**Affiliations:** 1 School of Chemical Engineering, University of Birmingham, Edgbaston, Birmingham B15 2TT, UK; cka329@student.bham.ac.uk (C.A.); t.miri@bham.ac.uk (T.M.); 2 Department of Chemical and Biological Engineering, The University of Sheffield, Sheffield S1 3JD, UK; Abarasi.Hart@sheffield.ac.uk

**Keywords:** bacteriocin, spores, food processing, food safety, antimicrobial peptides

## Abstract

Conventional thermal and chemical treatments used in food preservation have come under scrutiny by consumers who demand minimally processed foods free from chemical agents but microbiologically safe. As a result, antimicrobial peptides (AMPs) such as bacteriocins and nisin that are ribosomally synthesised by bacteria, more prominently by the lactic acid bacteria (LAB) have appeared as a potent alternative due to their multiple biological activities. They represent a powerful strategy to prevent the development of spore-forming microorganisms in foods. Unlike thermal methods, they are natural without an adverse impact on food organoleptic and nutritional attributes. AMPs such as nisin and bacteriocins are generally effective in eliminating the vegetative forms of spore-forming bacteria compared to the more resilient spore forms. However, in combination with other non-thermal treatments, such as high pressure, supercritical carbon dioxide, electric pulses, a synergistic effect with AMPs such as nisin exists and has been proven to be effective in the inactivation of microbial spores through the disruption of the spore structure and prevention of spore outgrowth. The control of microbial spores in foods is essential in maintaining food safety and extension of shelf-life. Thus, exploration of the mechanisms of action of AMPs such as nisin is critical for their design and effective application in the food industry. This review harmonises information on the mechanisms of bacteria inactivation from published literature and the utilisation of AMPs in the control of microbial spores in food. It highlights future perspectives in research and application in food processing.

## 1. Introduction

Food preservation is one of the major challenges in the food industry. This is because resistant bacterial spores are perfect vehicles for spoiling food and infecting humans. Hence, the fight against foodborne illness and food spoilage due to spore-forming bacteria has become a major public health problem. As a result, the inactivation of bacterial endospores is a critical step in the food processing industry to ensure consumer safety and stable shelf-life [1]. Micro-organisms produce several chemical agents as primary or secondary metabolites during their growth. These agents serve various functions related to the growth, metabolism of complex nutrients and to aid in competition. One of such chemical agents is the antimicrobial peptides (AMPs), also known as bioactive peptides, bacteriocins or antimicrobial activity peptides [2,3,4]. AMPs are assorted and abundant cluster of biomolecules and natural proteins domicile in animals, plants and bacteria responsible for defence of the host from pathogenic organisms [5,6]. As host defence peptides, AMPs can be classified as being cationic (positively charged) and amphiphilic (hydrophilic and hydrophobic) α-helical peptide molecules [7]. The methodology used in synthesising AMPs include chemical, enzymatic and recombinant techniques. They offer alternative to the chemical preservatives of foods to improve shelf-life. Presently, nisin is the only AMP extensively employed as a bio-preservative in food [5]. The utilisation of bio-preservatives offers compatibility than the use of chemical preservatives such as nitrites and sulphur dioxide, which could adversely impact on the quality and nutrition level of foods and on human health. Therefore, the major benefits the AMPs in the preservation of foods include no alteration of quality and it is not harmful [8]. The most widely studied classes of AMPs are those with antibacterial activity. This is due to their natural antimicrobial properties and a broad-spectrum of activity against bacteria, fungi, and viruses. In this regards, AMPs produced by bacteria, insects, amphibians and mammals, and those synthesised chemically are potential candidates for the design and development of new antimicrobial agents.

A wide range of bacteria synthesizes AMPs such as bacteriocins, which are potential alternatives to traditional antibiotics. These peptides have a high potency and a low toxicity, can be produced in situ by probiotics and can be bioengineered. However, the most predominant producers are the lactic acid bacteria (LAB) that easily break down lactose and other sugars to produce lactic acid, diacyl, hydrogen peroxide and other metabolites [9]. The production of bioactive peptides is an adaptation mechanism, aiding competition. These peptides exert a bactericidal or bacteriostatic effect against closely related strains of the producer organism and other bacteria genera [10]. Although, AMPs inhibit the growth of bacteria, they are not antibiotics. The difference between bacteriocins and antibiotics is that whilst antibiotics are secondary metabolites and enzymatically synthesized, bacteriocins are primary metabolites and are ribosomally synthesized [11]. Nisin is an example of AMPs produced by *Lactococcus lactis* with antimicrobial activity against several Gram-positive bacteria [12]. Nisin has shown low toxicity as wel as antibacterial activity, which proves its used as a food preservative [13].

**Figure 1:**
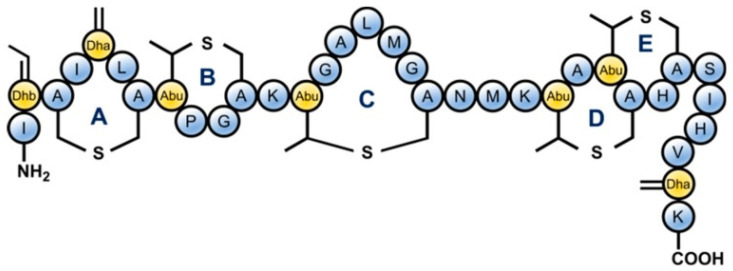
Structure of Nisin which is a lantibiotic AMP due to the presence of uncommon lanthionine rings in the structure and also the unsaturated amino acids introduced by posttranslational modifications: Dhb = dehydrobutyrine, Dha = dehydroalanine and Abu = aminobutyric acid [22].

In food processing and preservation, there is growing interest by consumers in minimally processed foods that have not been subjected to rigorous thermal treatment, free from chemical preservatives but still microbiologically safe. Food production that fits these criteria is a challenge in the food industry as thermal and chemical treatments are widely employed for food safety and shelf-life elongation. Thus, novel approaches have to be used to achieve these and control potential microbial agents such as the spore formers *Bacillus* sp. and *Clostridium* sp. Traditionally, *Bacillus* sp. and *Clostridium* spores are eliminated from foods using extreme treatments, including high temperature and chemicals. Although these treatments effectively eliminate bacteria spores, they negatively affect the nutritional and organoleptic quality of the foods [14,15]. The use of antimicrobial peptides can be an approach for controlling these food spoilage/poisoning organisms in minimally processed foods. APMs are generally classified into three, based on the producing strains, chemical composition, molecular weight, and action [16]. Class I AMPs contains the uncommon amino acid lanthionine and are enzymatically modified during biosynthesis, Class II AMPs are small and unmodified with a size of about 10 kDa, while the Class II are also unmodified but with a size larger than 10 kDa [17]. Currently, there are several characterized and purified bacteriocins. Some examples include; subtilin (Class I), thuricin (i.e., sactibiotic subclass of bacteriocin, class IId), cerein (class IIb sec-independent bacteriocin), and plantaricin (class IIa bacteriocin) processed for use as food additives/preservatives. However, the most common APM is nisin (class I bacteriocin) which is produced by *L. lactis* and is generally regarded as safe (GRAS) for use in food products [18,19]. Figure 1 shows the molecular structure of nisin a typical AMP. Nisin is a product of fermentation of food-grade bacterium, and has demonstrated a broad-spectrum of antibacterial activity including activity against many pathogenic bacteria that are responsible for food-spoilage. Hence, the safety and efficacy of nisin as a food preservative have resulted in widespread usage. Nisin is a known member of the lantibiotics AMPs, since it contains the uncommon amino acid lanthionine, with considerable potential in food preservation. However, only nisin has been satisfactorily characterized to be used for this purpose. For this reason, nisin has been approved by the United States Food and Drug Administration US-FDA [15]. Consequently, the combined effect of high pressure CO_2_ and nisin to inactivate *Bacillus subtilis* spores has been investigated and reported by Rao et al. [20]. It was discovered that the damaged spores coat and cortex by diffusing high pressure CO_2_ aided the penetration of nisin into the spores’ inner membrane, resulting in higher inactivation. This is in agreement with the result of Li and co-workers [21], where high-pressure CO_2_ was combined with 0.02% nisin at 10 MPa and 32 ℃ for 15 min produced a superior inactivation of *E. coli* and *Staphylococcus aureus* than application of high-pressure CO_2_ alone.

AMPs have the advantage of having a minimal impact on the organoleptic quality of foods without the degradation of the nutritional value which may occur with the use of some chemical preservatives [23]. Furthermore, they are easily digested by the human gastrointestinal tract; thus they do not get into the systemic circulation, reducing the possibility of having suboptimal levels in the body which can lead to the development of antimicrobial drug resistance [24,25]. Within food matrices, AMPs remain stable in wide pH and temperature ranges, retaining their antimicrobial activity after processing treatments that the foods receive [26]. This review focuses on the progress that has been made on the use of AMPs such as nisin to inactivate spore-forming bacteria and spores itself. It discusses the different types of AMPs, purification methods, mode of action and mechanisms of inactivating spores-forming bacteria and spores in order to preserve food and enhance shelf-life without impacting on quality and nutritional attributes.

## 2. Inactivation of Bacteria Spores by AMPs

Bacteria spores are abundant in the environment and can contaminate foods during different production processes [27]. Control of spores and spore-forming microbes in food is important because of their resilient nature, as their survivability in processed foods and subsequent germination under favourable conditions would shorten shelf-life, cause spoilage and food poisoning. In addition to food spoilage, foodborne illnesses resulting from toxin production [28], especially in canned foods. For instance, *Bacillus* and *Clostridium* species are known for producing emetic toxins, exo and neurotoxins, which are responsible for specific symptoms associated with the consumption of contaminated foods. The formation of bacterial toxins by *Clostridium* and *Bacillus* sp. is usually achieved by vegetative cells after germination of the spores and during growth [28]. Thus, for ensuring food safety, the spores of these bacteria genera need not be eliminated in totality but rather suppressed from germinating in foods. 

Generally, the control of spores and spore-forming bacteria is achieved traditionally by prolonged heating targeted at eliminating the vegetative forms of the organism to prevent spore formation. However, this method of preservation can negatively affect food quality (both nutritionally and food acceptance) and cannot be applied to some food products particularly proteinous foods which are heat sensitive. The alternative approaches, therefore, is to control, suppress and prevent the microbial spores in foods from emerging. This can be achieved with the aid of antimicrobial peptides, which has demonstrated potential inhibiting spores’ germination or outgrowth. This is usually achieved by the direct inclusion of AMP into the food or the fermentation of foods using bacteriocinogenic bacteria which utilises food substrate and synthesize the antimicrobial peptides directly into the food product. The functions of the AMPs depend not only their specific amino acid components and three-dimensional structure but also on their interfacial activity. The interfacial properties and the physicochemical interactions are important factors that control the biological activities of these AMPs with the membrane-destabilizing and membrane-permeabilizing abilities [7]. Hence, the amino acid composition, amphipathicity, helicity, cationicity and size enhance their insertion into lipid membranes, resulting in the inactivation of the target spore-forming microbes [29]. It is evident that AMPs possess some specific structure and features that allow them to interact with, bind to, and disrupt cell membranes. In 2013, Vicente et al. studied the interactions between a membrane mimetic and the cationic AMP Ctx(Ile^21^)-Ha, an equivalent consisting the paramagnetic amino acid 2,2,6,6-tetramethylpiperidine-1-oxyl-4-amino-4-carboxylic acid (TOAC) incorporated at residue positions n = 0, 2, and 13. It was observed with the aid of fluorescence experiments that all peptides were able to interact with lysophosphocholine micelles. 

In a study by Oman and van der Donk [30], haloduracin, a bacteriocin produced by *Bacillus halodurans* C-125 inhibited the germination and spore outgrowth of *Bacillus anthracis*. In another study by Martínez-Cuesta et al. [31], a lacticin 3147-producing *L. lactis* demonstrated the ability to control the outgrowth of *Clostridium* spores in semi-hard cheese. Other strategies in using antimicrobial peptides in food processing for the control of spores involve inoculation of foods with bacteriocinogenic lactic acid bacteria (LAB) for in-situ production of bacteriocins. This has been applied in the control of *Clostridium tyrobutyricum* [32], using AMPs-producing *Lactobacillus gasseri* K7 in cheese. These potent abilities of bacteriocinogenic LAB to inhibit and prevent the outgrowth of bacteria spores show that LABs, when utilized as a starter culture in fermentations, perform a dual role which includes microbial fermentation and food preservation. This is bearing in mind that many bacteriocinogenic LAB are originally isolated as part of food flora and are already responsible for wild type fermentations in these foods. Thus, when used within related food systems, they can result in food fermentations without undesirable metabolites. This approach has been employed in several studies to control mainly vegetative cells and spores as demonstrated in the study by Garde et al. [33]. They isolated *L. lactis* subsp. *lactis* INIA 415, a bacteriocinogenic LAB that produces nisin Z and Lacticin 481 from Manchego cheese and utilized in the fermentation of Hispanico cheese to develop volatile compounds and preservation [34]. In a subsequent study by Garde et al. [35], the outgrowth/germination of *Clostridium heijerinckii* spores was inhibited by AMP-producing lactic acid bacteria culture in ovine milk cheese. Compared to naturally fermented uncontaminated cheese samples, contamination of the cheese with *Clostridium heijerinckii* while fermenting with a bacteriocinogenic LAB resulted in a late blowing effect; thus, highlighting suppressed sporulation of the vegetative cells [35]. Similar sporicidal and sporostatic effect of APMs such as nisin has been reported in earlier studies [36]. Table 1 displays some AMPs that have proven effective against bacterial spores. Their antimicrobial mechanisms are different from those of traditional antibiotics. The mechanism of action is based on the ability of AMPs to alter membrane permeability and promote discomposure in the spore-forming microbe cell membrane. Furthermore, they can act on different targets in the cells such as DNA, RNA, regulatory enzymes, and other proteins [37].

To date, only nisin and pediocin PA-1 have been satisfactorily characterized to be used in the food industry as bio-preservatives [46]. In other words, there is need to sufficiently well characterise other AMPs for use in food preservation.

## 3. Mechanism of Microbial Inactivation by AMP Nisin

An understanding of AMPs and their mechanisms of action will provide insight into the development and design of the next generation synthetic and efficacious AMPs for application in food preservation. When AMP interact with bacterial membranes, there are at least nine hypotheses of mechanisms of action reported in the literature, which include: (1) electroporation; (2) carpet model, (3) membrane thinning or thickening, (4) non-lytic membrane depolarization, (5) toroidal pore, (6) oxidized lipid targeting, (7) barrel stave, (8) disordered toroidal pore, and (9) non-bilayer intermediate [6,7,47]. However, cationic AMPs can bind and interact with the negatively charged bacterial cell membranes, resulting in the change of the electrochemical potential, which can cause cell membrane damage and the permeation of larger molecules such as proteins. This destruction of the cell morphology and membranes eventually leads to the cell death or spore’s inactivation [7]. It has been reported that the lantibiotic nisin inactivates growth of vegetative spore-forming Gram-positive bacteria by binding to lipid II, disrupting cell wall biosynthesis and facilitating the formation of pores [48]. Many in vitro studies have shown the therapeutic effectiveness of nisin, particularly for the control of antibiotic-resistant bacteria strain. 

Unlike vegetative cells, it is well known that the spore’s endospore structure (i.e., cortex and coat) provides protection in a widespread range of environments including protection against oxidation, radiation, desiccation, heat, and extremes in pH [15]. Therefore, inhibition of spore outgrowth and membrane disruption could result in inactivation of spore-forming bacteria, leading in shelf-life elongation [15]. The mechanism of microbial inactivation by AMPs also involves the degradation of the cell membrane and pore formation, resulting in the leaching out of cellular materials and cell death [49]. These actions are less likely to induce resistance, which is a major problem associated with the use of conventional antibiotics in microbial inactivation. This is because bacteriocin fragments do not interact with the target cells [10]. However, with regards to microbial spores, the mechanism of inactivation is not fully elucidated, as precious studies suggested either covalent binding to a spore target or loss of membrane integrity such as disruption of cell wall biosynthesis via binding to lipid II is yet to be completely investigated. Anti-spore activities of AMPs such as bacteriocins and nisin have been demonstrated against both the vegetative cells and spores of spore-forming bacteria [40,44,45]. They either inhibit spore germination in dormant spores or inhibit spore outgrowth in germinating spores [40,50,51]. However, some bacteriocins such as nisin cannot interfere with spore germination but instead exert their inhibitory/inactivation activity after spore germination [52], by binding to lipid II in the germinating spores (which is absent in dormant spores). This binding prevents the cells after germination from becoming metabolically active thus inhibiting the establishment of membrane potential and oxidative metabolism, ultimately leading to cell death [53]. 

**Figure 2:**
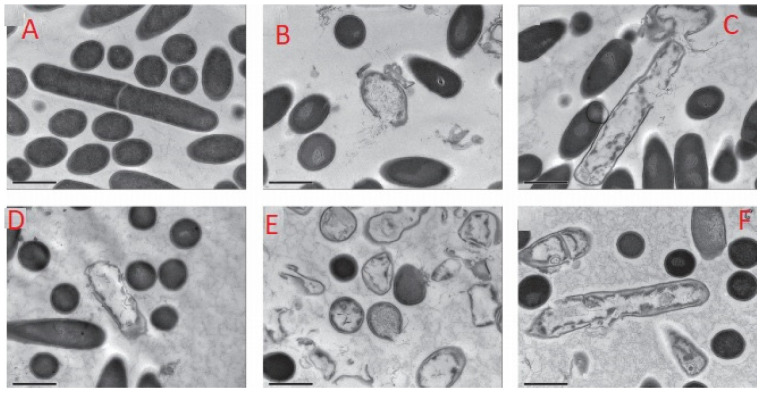
Transmission electron micrographs of *C. difficile* ATCC 630 grown in brain heart infusion (BHI) broth and treated in suspension (10^6^ c.f.u.) for 1 min with (**A**) no inhibitor, (**B**) nisin A (16 µg/mL), (**C**) nisin Z (64 µg/mL), (**D**) vancomycin (5 µg/mL), (**E**) nisin A (32 µg/mL) and (**F**) nisin Z (128 µg/mL) [54].

Figure 2 shows transmission electron micrographs of the effect of treatment of *Clostridium difficle* spores and vegetative cells treated with nisins concentration in the range of 0–3.2 μg/mL and the antibiotic vancomycin. In the study, nisin Z obtained from culture of *L. lactis* subsp. *lactis* biovar. *diacetylactis* was tested along with commercial nisin A. It was found that both nisin A and Z inhibited the growth of all *Clostridium difficile* isolated. Untreated cells without nisin showed a completely intact cell structure (Figure 2A). Whereas, nisin treated cells exhibited disruptions in the cell membrane and release of cytoplasmic contents, resulting in their inactivation (Figure 2B–F). It is believed that the inactivation is as a result of the antibacterial activity of nisin, causing membrane pore formation and interfering with cell wall biosynthesis. These findings are consistent with Gut et al. [53], they studied the inhibition of *Bacillus anthracis* spore outgrowth by the lantibiotic nisin.

It was found that in the presence of nisin, germination decreased by 0.3 ± 0.08 and 0.8±0.4 log corresponding to concentrations of 0.1 and 0.2 μg/mL. However, at a higher concentration 0.4 μg/mL a significant decrease in germination greater than 1.8 log was observed. The *C. difficile* spores were completely inhibited at a concentration of ≥12.8 μg/mL of nisin [54]. In summary, the inhibitory mechanism of nisin on the outgrowth of spores can be attributed to the combination of binding lipid II and membrane disruption [53,54]. In other words, the interactions of AMPs with anions on the surface of microbial membranes play crucial roles in the inactivation. Although, cell membrane disruption has been reported as a mechanism of inactivation by AMPs, there is presently no single theory or mode that can be applied to explain the mechanism of action of all AMPs as most studies uses artificial lipid membranes. This is because the inactivation mechanism is multifaceted. 

The resilience of spores orchestrated by the presence of dipicolinic acid, make them more difficult to be eliminated [55]. Hence, to ensure complete inactivation of spores, AMPs has been applied in combination with emerging food processing technologies such as pulse electric field (PEF), moderate heat treatment, ultrasound technique and supercritical carbon dioxide [15,56]. In light of this, a synergistic sporostatic/sporicidal activity of the AMPs nisin and lysozyme has shown to be effective in-vitro against the vegetative cells of *Clostridium difficle* [57], while a recent study by Chai et al. [58], showed enhanced inactivation of spore outgrowth using this synergistic approach. In 2016, Rao et al. reported the synergistic effect of combining supercritical carbon dioxide and nisin to inactivate *Bacillus subtilis* spores [20]. The findings show that supercritical carbon dioxide enhanced the penetration and effectiveness of the nisin resulting complete inactivation. Similarly, Galvagno et al. [59], recorded a 3-log reduction in the germination of *Bacillus megaterium* spores in beer after treatment with a combination of nisin and PEF technology at 60 °C. Also, combined treatment of foods with AMPs and heat has been shown to bring about effective inhibition of bacteria spores. This can be attributed to the impact of heat on the permeability of the endospore, allowing a greater activity of the bacteriocin AMP. A study by Lucas et al. [42], inoculated tomato paste, canned pineapple juice and syrup from canned peaches with spores of *Bacillus coagulans* CECT. It was observed that application of the bacteriocin enterocin AS-48 to the foods was only effective in inhibiting the vegetative cells of the organism and not the spores. A marked reduction in viable counts was recorded when in the presence of enterocin AS-48, the food was subjected to a 5-min heat treatment from 80 °C to 95 °C compared to foods that were subjected to only heat treatments or treatment with enterocin AS-48. A similar result was obtained when *Bacillus cereus* spores were subjected to mild heat treatment in the presence of enterocin AS-48 [60]. More also, high hydrostatic pressure (HHP) and the addition of nisin has been studied for inactivation of spores of *Bacillus cereus* ATCC 9139 inoculated in model cheeses made of raw milk [61]. At a pressure of 400 MPa and temperature 30 °C, the highest inactivation (2.4 ± 0.1- log_10_ cfu/g) was achieved in the presence of nisin concentration of 1.56 mg/L of milk. Similarly, an investigation of the sensitivity of *Paenibacillus* sp. and *Terribacillus aidingensis* spores to combined action of high pressure (HP), nisin and moderate heating at 500 MPa/10 min/50 °C showed 6log (CFU/mL) and 4log (CFU/mL) reduction, respectively [62]. In this case, the pressurization may inflict sublethal damage to both gram-positive and gram-negative spore-forming bacteria cells, making them more susceptible to AMP molecules such as nisin. This shows that the efficiency of AMP such as nisin against spores is enhanced when used in conjunction with other treatment technologies; however, the technique must demonstrate minimal impact on food sensory and nutritional properties. This synergistic combination could improve food shelf-life and safety as well as compensate for the limitations of a single process. In recent times, Modugno et al. [63], has demonstrated the efficacy of high pressure in the sensitization of heat resistant spores of *Bacillus pumilus, B. sporothermodurans, B. licheniformis, B. weihenstephanensis*, and *Clostridium* sp., making them susceptible to the AMPs nisin.

In 2019, Fan et al. evaluated the synergetic inhibitory effects of ultrasound and nisin/carvacrol on spore germination, outgrowth and proliferation of the vegetative cells of *Bacillus subtilis* [64]. According to Hofstetter et al. nisin inhibited cell growth and inactivated endospore in three *Clostridium* species at high temperature (90 °C) and high pressure (600 MPa) [38]. The effect of nisin on endospore inactivation in the *Clostridium* species was found to be specie dependent. The minimum inhibitory concentration of the nisin on different *Clostridium* species are; *Clostridium difficile* 3195 (1.09 ± 0.38 mg/L), *Clostridium sporogenes* ATCC 7955 (1.11 ± 0.48 mg/L), and *Clostridium beijerinckii* ATCC 8260 (3.47 ± 1.50 mg/L), respectively. The optimization parameters for nisin inactivation of *Clostridium perfringens* at high temperature and high pressure was estimated experimentally by Gao et al. [65], to be at a pressure of 654 MPa (pressure holding time of 13.6 min) and temperature of 74 °C, and a nisin concentration of 328 IU/mL. Furthermore, Le Lay et al. [54], reported *Clostridium difficile* spores to be sensitive to nisin A at 25.6 µg/mL which was observed to reduce the spore viability by 50%. Rodgers et al. [66], found *Clostridium botulinum* spores to be sensitive to nisin at 50–100 IU/mL (60.24–120.48 μg/mL) and pediocin at 10–20 AU/mL. Gut et al. [67], recorded the inhibitory effect of nisin on *Bacillus anthracis* spore with IC_50_ and IC_90_ of 0.57 µM and 0.90 µM, respectively. An effective synergistic combination of nisin and osmotic pressure against *Clostridium difficile* spore was reported by Nerandzic and Donskey [39]. A combination of heat, acidification and nisin at 90 °C, pH 4.5 and 500 IU/g (602.41 μg/mL) respectively resulted in the inactivation of *Clostridium sporogenes* spores [68]. *Clostridium botulinum* spores were inactivated by nisin with a combination of high pressure and temperature pressure at a pressure of 545.0 MPa, a temperature of 51 °C, a pressure holding time of 13.3 min and nisin concentration of 129 IU/mL (155.42 μg/mL) respectively [69]. Ros-Chumillas et al. [70], suggested the use of nisin and a thermal treatment the control of *Clostridium sporogenes* spores. From their study, nisin alone had no effect on bacterial spores, but after a 100 °C thermal treatment for 3 s, there was inactivation of spores. Hence, the need for synergistic effect in combination with other emerging non-thermal food processing techniques. Aouadhi et al. [50], concluded that the use of high temperature and nisin enhance the effectiveness of the use of high pressure in the inactivation of heat resistant spores of *Bacillus sporothermodurans*. A study on the combined effect of temperature and nisin was also carried out by Aouadhi et al. in 2014 using the spores of *Bacillus sporothermodurans* in water, skim milk and chocolate milk. They concluded a temperature range of 73 to 106 °C and nisin concentration of 15 to 184 IU/mL for inactivation of *Bacillus sporothermodurans* spores. In distilled water, 95 °C, 125 IU/mL for 12 min, in skim milk 100 °C, 134 IU/mL for 13 min and in chocolate milk 100 °C, 135 IU/mL for 15 min. However, it has been reported that the effectiveness of nisin is often affected by environmental factors such as pH, temperature, food composition, structure, as well as food microbiota [46,48].

## 4. Impact of AMPs on Food Quality, Advantages and Challenges in Food Processing

AMPs have been used in the preservation of a variety of liquid and solid foods. They are usually employed either alone or in hurdle technology (i.e., in a combination with other preservative methods) [71]. Aside their chemical and enzymatic stability in foods, they have proven to offer beneficial effects such as improving the quality and flavour of foods [72]. Thus, in addition to its preservative role, AMPs such as bacteriocins and nisin have been employed to enhance the quality of final products, including accelerated cheese ripening and flavour development [73]. The improvement in the organoleptic quality of foods following treatment with bacteriocins is a beneficial attribute compared to some chemical preservatives that leave artificial tastes in food substances. Oshima et al. [74], found that when using low-temperature treatment to retain aroma and flavour in food, nisin A is effective as a natural preservative in preventing bacteria spoilage of high-fat milk pudding, thereby extending its shelf life, without compromising food safety. Nisin improved the shelf life of cooked potato products and inhibited *Bacillus* sp. and *Clostridium* sp. [75]. Due to the broad spectra of inhibition by AMPs, it is obvious their use in the food industry could help reduce the addition of chemical preservatives as well as the intensity of thermal treatments, resulting in foods that are more naturally preserved. In the food industries, chemical preservatives including curing salts, sodium, nitrate, nitrite and potassium are employed for microbial inhibition, colour fixation and delayed lipid oxidation, amongst others. These chemicals, besides their beneficial effects, could have harmful health consequences upon excessive consumption. Thus, AMPs offer a safe alternative for their usage. Furthermore, the AMPs such as nisin have the advantage of remaining active over a wide temperature, pH, salt and solute concentrations [26]. The major drawback to the use of AMPs in food processing is their narrow spectrum of activity which makes them generally ineffective against other non-related microorganisms which can be pathogenic in foods, the relatively low solubility of some bacteriocins including nisin and the possibility of enzymatic degradation of the peptides [76]. These attributes make the application of the bacteriocins limited for food preservation or, at best, to be used only as one component in the hurdle concept for food preservation. The continued application of AMPs such as nisin in food preservation, especially for the control of bacterial spores in foods will depend on increasing their solubility in foods to allow for greater activity/efficacy. Also, the growing concerns and the occurrence of resistance bacteria, means it is worth investigating the bactericidal efficacy of nisin in food preservation under the prevailing circumstance [46]. Likewise, the mechanisms of action resulting in inactivation spores and spore-forming bacteria by AMPs such as nisin is complicated and may differ among strains.

There is a disagreement in the activity of bacteriocin on bacterial spores. Some researchers report that bacteriocin does not affect spore or increase their susceptibility to heat but only act against germinating spores. Other researchers claim that bacteriocin increases the sensitivity of heat resistant spores to heat treatment. This disparity in the report needs to be carefully examined. Pei et al. [43], reported that bacteriocin RC20975 is not sporicidal to the spores of *Alicyclobacillus acidoterrestris* in apple juice but contributed to a reduction in thermal resistance of the bacterium. Wandling et al. [77], suggested that nisin in skim milk is likely to cause heat sensitivity in bacterial spores and prevent outgrowth of survived spores. Spores of *Bacillus anthracis* Sterne exposed to nisin were 10 times sensitive to pressure treatment than unexposed spores [78].

Mansour et al. [79], stated that nisin does not affect spores before germination. This is supported by an earlier report by Campbell and Sniff [80], which concluded that nisin and subtilin do not reduce the thermal resistance of *Bacillus coagulans* spores; they continued to state that nisin and subtilin are neither sporicidal nor sporostatic but inhibit only the outgrowth of germinated spores. Nisin at 50 UI/mL inhibited bacterial spore outgrowth but didn’t affect their germination [40]. Pol et al. [56], reported that nisin and pulse electric field, PEF (i.e., non-thermal food processing technique using short, high voltage pulses) did not inactivate spore or increase heat sensitivity of *Bacillus cereus* spores rather nisin and PEF were found to affect germinating spores. Nisin was better than PEF (whose activity was lost after 50 min and did not inactivate all germinated spores). Nisin was found to be effective at the same level as heat treatment. From Mansour et al. [79], 25 IU/mL of nisin inhibited spore outgrowth of *Bacillus licheniformis* for 10 h before regrowth, but a combination of 30 IU/mL of nisin and 100 mg/mL of monolaurin resulted in total spore growth inhibition at a pH of 6.0. Bacteriocin AS-48 inhibited growth and sporulation in *Bacillus cereus*; intact spore was resistant but became sensitive at germination [41].

## 5. Conclusions and Future Perspectives

This study reports the application of antimicrobial peptides (AMPs) in food preservation because of their ability to inactivate spores and spore-forming bacteria. The review focused more on nisin, which is one of the sufficiently characterised AMPs for use in the food industry. The mechanisms of spore-forming bacteria inactivation, the effectiveness of using nisin AMP alone and the synergistic effect of combining AMPs with other non-thermal emerging technologies were explored. The mechanisms of inactivating spore-forming bacteria can be summarised as thus membrane disruption, holes formation resulting in the release of cellular materials and enzymes and cellular material deactivation. It was found that antimicrobial peptides’ efficacy in inactivation of spores can be enhanced by any treatment that allows for more permeability into the spore walls and membranes such as coat and cortex. However, the effectiveness and efficiency of inactivation is enhanced by combining AMPs with non-thermal techniques but must exert negligible effect on the food sensory and nutritional attributes. This is instructive for the canning industry, where the problem of spore germination in canned food poses a serious challenge resulting in spoilage and commercial losses. For an optimal application of nisin and other antimicrobial peptides in food preservation with a specific target on spores, different combinations of treatments need to be explored to inactivate these spores while having minimal impact on the organoleptic qualities of food products. Likewise, the growing concerns of resistance bacteria, means the bactericidal efficacy of nisin and other AMPs in food preservation require further examination. In future studies, modern characterisation and molecular biology techniques would offer a new opportunity to gain insight into the mechanisms of inactivation and improve design and deployment of AMPs. The analysis of literature has shown that further study on nisin resistance will not only result in comprehensive understanding of the characteristics of nisin but would also help improve the optimal conditions for the application of nisin in food preservation. Also, the application of bioengineering techniques to enhance antimicrobial activity and the spectrum of nisin activity, alongside its physicochemical properties such as heat stability, solubility and diffusion.

## Figures and Tables

**Table 1:** Some examples of AMPs with activity against bacteria spore formers.

AMPs	Spore Former	References
Nisin	*C. perfringens, C. sporogenes, C. botulinum, C. difficile, C. beijerinckii*	[38,39,40]
Enterocin	*A. acidoterrestris, B. cereus, B. licheniformis, G. stearothermophilus*	[41,42]
Bificin	*A. acidoterrestris*	[43]
Lacticin	*C. tyrobutyricum*	[31]
Plantaricin	*C. sporogenes*	[44]
Thurincin	*B. cereus*	[45]

## Data Availability

Not applicable.
